# Experimental *Plasmodium vivax* infection of key *Anopheles* species from the Brazilian Amazon

**DOI:** 10.1186/1475-2875-12-460

**Published:** 2013-12-21

**Authors:** Claudia M Rios-Velásquez, Keillen M Martins-Campos, Rejane C Simões, Thiago Izzo, Edineuza V dos Santos, Felipe AC Pessoa, José BP Lima, Wuelton M Monteiro, Nágila FC Secundino, Marcus VG Lacerda, Wanderli P Tadei, Paulo FP Pimenta

**Affiliations:** 1 Instituto Leônidas e Maria Deane, Fundação Oswaldo Cruz - Amazonas, Rua Teresina, 476, Adrianópolis, 69057-070 Manaus, AM, Brazil; 2 Instituto Oswaldo Cruz, Fundação Oswaldo Cruz, Rio de Janeiro, Brazil; 3 Instituto Nacional de Pesquisas da Amazônia, Manaus, AM, Brazil; 4 Ministerio da Saúde, Núcleo Amazonas/Fundação de Vigilância em Saúde, Manaus, AM, Brazil; 5 Fundação de Medicina Tropical Dr. Heitor Vieira Dourado, Manaus, AM, Brazil; 6 Universidade Federal de Mato Grosso, Cuiabá, MT, Brazil; 7 Universidade do Estado do Amazonas, Manaus, AM, Brazil; 8 Centro de Pesquisas René Rachou, Fundação Oswaldo Cruz-Minas Gerais, Av. Augusto de Lima, 1715, CEP 30190-002 Belo Horizonte, MG, Brazil

**Keywords:** *Plasmodium vivax*, *Anopheles*, Malaria, Membrane feeding assay, Infection rate, Oocysts

## Abstract

**Background:**

*Anopheles darlingi* is the major malaria vector in countries located in the Amazon region. *Anopheles aquasalis* and *Anopheles albitarsis s.l.* are also proven vectors in this region. *Anopheles nuneztovari s.l.* and *Anopheles triannulatus s.l.* were found infected with *Plasmodium vivax;* however, their status as vectors is not yet well defined. Knowledge of susceptibility of Amazon anopheline populations to *Plasmodium* infection is necessary to better understand their vector capacity. Laboratory colonization of *An. darlingi*, the main Amazon vector, has proven to be difficult and presently *An. aquasalis* is the only available autonomous colony.

**Methods:**

Larvae of *An. darlingi, An. albitarsis s.l., An. nuneztovari s.l.* and *An. triannulatus s.l.* were collected in the field and reared until adult stage. Adults of *An. aquasalis* were obtained from a well-established colony. Mosquitoes were blood-fed using a membrane-feeding device containing infected blood from malarial patients.

The infection of the distinct *Anopheles* species was evaluated by the impact variance of the following parameters: (a) parasitaemia density; (b) blood serum inactivation of the infective bloodmeal; (c) influence of gametocyte number on infection rates and number of oocysts. The goal of this work was to compare the susceptibility to *P. vivax* of four field-collected *Anopheles* species with colonized *An. aquasalis.*

**Results:**

All *Anopheles* species tested were susceptible to *P. vivax* infection, nevertheless the proportion of infected mosquitoes and the infection intensity measured by oocyst number varied significantly among species. Inactivation of the blood serum prior to mosquito feeding increased infection rates in *An. darlingi* and *An. triannulatus s.l.,* but was diminished in *An. albitarsis s.l.* and *An. aquasalis*. There was a positive correlation between gametocyte density and the infection rate in all tests (Z = −8.37; p < 0.001) but varied among the mosquito species. *Anopheles albitarsis s.l., An. aquasalis* and *An. nuneztovari s.l.* had higher infection rates than *An. darlingi*.

**Conclusion:**

All field-collected *Anopheles* species, as well as colonized *An. aquasalis* are susceptible to experimental *P. vivax* infections by membrane feeding assays. *Anopheles darlingi, An. albitarsis s.l.* and *An. aquasalis* are very susceptible to *P. vivax* infection. However, colonized *An. aquasalis* mosquitoes showed the higher infection intensity represented by infection rate and oocyst numbers. This study is the first to characterize experimental development of *Plasmodium* infections in Amazon *Anopheles* vectors and also to endorse that *P. vivax* infection of colonized *An. aquasalis* is a feasible laboratory model.

## Background

Malaria is an infectious disease with major impact on global public health and economy. Currently, malaria threatens almost one third of the world population in 103 tropical countries, resulting in 207 million of clinical cases and 627,000 deaths in 2012 [1]. In the Americas, 21 countries are affected by malaria, with approximately 1.1 million cases in 2010, most of which occurred in the Amazon basin, which covers 40% of South American territory. In Brazil, the Federal Health System Surveillance registered 267,000 cases in 2011, most of them (99.8%) in the Amazon region [2].

Human malaria parasites in the genus *Plasmodium* are transmitted by anopheline mosquitoes. *Plasmodium falciparum* and *Plasmodium vivax* are the main species-affecting humans. Actually, since the mid-1990s, *P. vivax* has become the predominant New World malaria species, as it has expanded its range throughout South America [1]. In Brazil, 84% of registered malaria cases are caused by *P. vivax*[2]. The consequences of this increase in distribution are higher exposure and increased infection, adding to difficulties in controlling the disease. Although *P. vivax* malaria is generally considered to be relatively benign, there are numerous reports of complex cases with severe clinical complications and deaths [3-10].

One keystone stage in the *Plasmodium* life cycle is the infection of mosquito vectors. Among the 33 *Anopheles* mosquito species described from the Brazilian Amazon region, *Anopheles darlingi* is considered to be the main malaria vector. Other anopheline species can be considered secondary or occasional malaria vectors because of their population density, anthropophilic behavior and natural infectivity across their geographic distributions [11-14]. *Anopheles albitarsis s.l., An. nuneztovari s.l.* and *An. triannulatus s.l.* are commonly collected in the Amazon, and they have been observed infected with *P. vivax* and *P. falciparum*, but their role as malaria vectors has not yet been elucidated [15-25]. *Anopheles aquasalis* is distributed predominantly along the Atlantic coast because of its tolerance to saltwater environment and has been found naturally infected by *P. vivax*[20,26-28] in the Eastern Amazon region.

Outside the Brazilian Amazon, *An. darlingi* has been associated with malaria transmission in Bolivia, Colombia, French Guiana, Guyana, Peru, Suriname, and Venezuela [12,29]; *An. albitarsis s.l.* in Venezuela [30]; *An. nuneztovari s.l.* in Venezuela [30], Peru [31] and Colombia [32,33]; *An. triannulatus s.l.* in Venezuela [34] and Peru [31]; and, *An. aquasalis* in Trinidad [35], Guyana [28] and Venezuela, where it is considered to be the primary coastal malaria vector of *P. vivax*[36].

The life cycle of *Plasmodium spp.* starts when mosquitoes ingest gametocytes, the parasite sexual stage, during the blood meal taken through the skin of infected vertebrate hosts. Inside the mosquito alimentary tract, fusion between male and female gametocytes produces motile ookinetes, which traverse the mosquito midgut epithelium to form oocysts [37,38]. The presence of well-developed oocysts outside mosquito midgut indicates *Plasmodium* establishment in a susceptible vector and this parameter is used to determine the infection rate of a mosquito population [39,40].

In the field studies, the infection rate, i.e., the amount of individuals in a mosquito population that carry well-developed *Plasmodium* oocysts, is an important parameter for defining vector competence and thus, a key indicator in the description of malaria dynamics and transmission biology in a given geographic region. Indeed, infection rates in mosquito vectors are related to gametocyte survival, viability and success of fertilization, and finally, midgut invasion by the resulting ookinetes. However, not all gametocytes that are ingested by susceptible mosquito vectors reach the ookinete stage [41-43]. Factors such as gametocyte density, gender ratio and maturity, presence of anti-malarial drugs, human and mosquito immune factors, and intrinsic parasite factors influence gametocyte viability, fusion and infectivity and consequently oocyst formation [44-48]. To complete the sexual development of *Plasmodium* in the mosquito, sporozoites are released from the oocyst and go on to invade the salivary glands. Once the salivary glands become infected with sporozoites, the mosquito is infectious to humans during the next blood meal [49-51].

It is well known that among the over 400 species of mosquitoes in the genus *Anopheles* only about 10% are important as vectors of human malaria. There is a multitude of both ecological and genetic determinants that influence vector competence, both among species and even at the level of geographic populations within a single species [41-45,47]. Differences in susceptibility to *Plasmodium* infection among the putative vectors of malaria in the Amazon have never been fully and carefully considered until this study.

Mosquito vectors of malaria from Africa and Asia have been well established in colonies and are feasible to maintain in laboratory. For example the *Anopheles gambiae*, the major vector in several African countries, is the most well studied mosquito, including its interaction with human and murine *Plasmodium* species causative agents of malaria [52,53]. Distinctly, the colonization of *An. darlingi*, the major Amazon vector, has proven to be difficult as well as other anopheline species from the region, and presently there is only *An. aquasalis* as an available autonomous colony. *Anopheles aquasalis* has been reared in laboratory as free mating since 1995 [54-56] and recently adapted as a well-established colony in Amazon institutions for experimental studies [57-59].

The goal of this work was to compare the susceptibility to *P. vivax* of colonized *An. aquasalis* with four groups of field-collected *Anopheles* species. This study was focused on vector infection rates as defined by the presence, quantification of oocysts and proportion (percentage) of infected individuals in these mosquito populations, following exposure to blood obtained from infected human patients. In addition, *P. vivax* infection in the vector was correlated with gametocyte numbers present in the circulating blood of infective human hosts. In laboratory studies, the infection rate is a critical part of the determination of vector competence. It is important to study *P. vivax* infection of New World vectors due to the huge gap regarding the knowledge comparing other vector-parasite pairs from the Old World.

## Methods

### Blood collection and ethic statements

Adult volunteers (ages >18 years) residents from the region of Manaus (State of Amazonas, Brazil) with *P. vivax* malaria infection diagnosed by blood smears were invited to participate in the study. Volunteers signed informed consent documents as blood sample donors. About 3 ml of blood samples were collected by venipuncture from volunteers and placed into a sterile lithium heparinate vacutainer tube. After blood collection, all patients were treated at the Fundação de Medicina Tropical Dr Heitor Vieira Dourado (located in the city of Manaus) or in the health posts from the region of Manaus where they were diagnosed, following ethical procedures determined by the Brazilian Health Ministry. This study was approved by the Brazilian National Ethics Committee Board (CONEP, 3726).

### *Plasmodium vivax* peripheral parasitaemia and gametocyte counts

Thick and thin blood smears from malarial patients were prepared by Giemsa staining method and examined under light microscope x100 oil immersion lens to confirm the presence of *P. vivax* parasites. Sexual (gametocyte) and asexual stages counting per 500 leukocytes were performed.

### *Anopheles* collections

Mosquito larvae were collected during one year at different breeding sites near the city of Manaus, capital of Amazonas State, Brazil: Puraquequara Road (Portela 03°03′16.4″S 59°53′44.0″W; Km 9 Vicinal 03°03′09.1″S 59°52′12.6″W; Carlão 03° 02′ 46.33000″ 59° 52′ 53.90000″); Brasileirinho Road (Raifram 03°02′09.5″S 59°52′15.5″W; Cristo Vive 03°01′33.1″S 59°51′07.7″W). Larvae were reared in the insectary as described elsewhere [17]. Emerged adult mosquitoes were identified as the following species: *An. darlingi*, *An. albitarsis s.l., An. nuneztovari s.l.* and *An. triannulatus s.l.*, as described elsewhere [60,61]. Field mosquitoes from each species were separated and housed in the rearing containers. *Anopheles aquasalis* mosquitoes originated from a colony established in 1995 [54] were reared from eggs to adult. All mosquitoes were *ad libitum* fed 10% sugar solution and kept in laboratory conditions at 26-28°C and 70-80% RH (relative humidity). Three- to five-days old adult females were used in all experiments. Pinned voucher specimens were deposited at the Biological Collection at the Instituto Leônidas e Maria Deane (Fiocruz, Amazonas)*.*

### *Plasmodium vivax* infection of mosquitoes via membrane feeding assay

Adult mosquitoes were sugar starved overnight prior to infection via membrane feeding assay. Individuals from each of the five species were separated in two experimental groups. One group was offered whole blood (WB) from *P. vivax* patients for a period of 45 to 90 minutes via membrane feeding assay (glass device covered with Parafilm®). Blood was held at 37-39°C through a hose system connected to a thermal bath. The second group was treated in similar way but with inactivated-blood serum (IBS). The *P. vivax* infective blood samples were centrifuged for 15 minutes at 2,000 g and the serum removed and heated for 1 hour at 56°C. Then, the inactivated serum was added back to the red blood cells containing parasites, suspended and offered to the mosquitoes. After the infective blood meals only fully engorged mosquitoes were transferred to rearing containers and maintained in the insectary as described above for the development of infection.

### Evidence of infected mosquitoes

Five to eight days after infective blood meal, midguts from the experimentally infected mosquitoes were dissected in phosphate buffered saline, stained with 2% commercial Mercurochrome (Merbromin), placed under a coverglass and examined for the presence of oocysts. The number of oocysts on the mosquito midgut was recorded.

### Data analysis

In this study, the blood-feeding rate was calculated as the proportion of female mosquitoes that were fully engorged after a blood meal. The susceptibility of the *Anopheles* species to *P. vivax* was evaluated by the presence and the number of oocysts in the midguts. The population infection rates were calculated by dividing the number of infected mosquitoes (those with one or more oocysts) by the number of dissected mosquitoes. G tests were used to compare the frequency of infection among all the studied mosquito species conjointly, as well as pairwise comparisons between each pair of species. The Kruskal-Wallis test evaluates differences in the number of oocysts between the infected mosquito species. Only positively infected mosquitoes were used for this last analysis. Conover-Inman test, *a posteriori*, was used for comparison of the number of oocysts among all studied mosquito species [62,63]. For each mosquito species, logistic regressions were used in order to evaluate if the probability of the mosquito to be infected was related to the number of gametocytes in the blood meal. Spearman’s Rank correlations were used in infected mosquitoes to correlate blood-circulating gametocyte numbers with oocyst numbers. G tests were used to compare the number of oocysts per infected midgut with the gametocytes present in the infective blood. G test was also used to evaluate if blood factors affect the mosquito infection probability, comparing the mosquito infection rate of the species fed on WB or ISB blood samples. All statistical analysis used α = 0,05 and the R-Project software version 2.13.1 (R Core Team).

## Results

**Figure 1:**
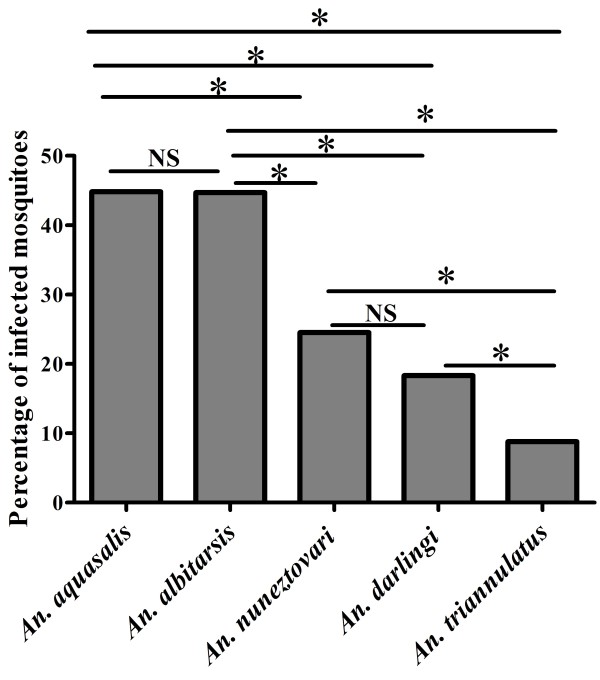
**Comparison of the susceptibility of WB-infected *Anopheles *** **from the Brazilian Amazon to *Plasmodium vivax *** (Bars with asterisk indicates significant pair comparisons between the species, * = p <0.01, NS = not significant).

A total of 2,449 adult female mosquitoes and 62 *P. vivax* isolates from malarial patients were used for the different experimental feeding assays and only the fully engorged mosquitoes after blood feeding were analyzed in this study (Table 1). The five mosquito species differed in regard to feeding time until engorgement. *Anopheles aquasalis, An. darlingi* and *An. triannulatus s.l.* fed the most rapidly with 64% of individuals fed to repletion in 40 minutes. *Anopheles albitarsis s.l.* and *An. nuneztovari s.l.* fed more slowly, with 72% and 64%, respectively, fully engorged over a period of approximately 60 to 80 minutes. The proportion of infected mosquitoes following engorgement on an infected blood meal was significantly different among species (G = 199.1, GL = 4, p <0.001) (Figure 1). *Anopheles aquasalis* and *An. albitarsis s.l.* showed very similar infection rates (G < 0.01, GL = 1, p = 0.98). Actually, *An. aquasalis* showed the highest infection rate (44.8%, remaining comparisons, G > 18, GL. = 1, p < 0.001) followed by *An. albitarsis s.l.* (44.7%), which were significantly different to all other mosquito species (G > 8.14, GL = 1, p < 0.01). *Anopheles nuneztovari s.l.* held the third highest infection rate (24.5%), and it was not statistically different to *An. darlingi,* which had an infection rate of 18.3% (G = 2.1, GL = 1, p = 0.148), but was different to *An. triannulatus s.l.,* with only 8.8% of the individuals infected (G = 14.6, GL = 1, p < 0.001). *Anopheles darlingi* also had a significantly higher infection rate compared with *An. triannulatus s.l.* (G = 13.1, GL = 1, p = 0.001) (Figure 1).

**Table 1:** **Infection rate and mean number of oocysts produced in *Anopheles *** **species from the Brazilian Amazon infected with whole blood (WB-infected mosquitoes) or inactivated blood serum (IBS-infected mosquitoes)**

***Anopheles *** **species**	**Number of gametocytaemic samples**	**Number of dissected gut**	**Infection rate (%)**	**Mean number of oocysts (Min –Max)**
	**WB-infected mosquitoes**			
*An. albitarsis* s.l.	29	861	44,8	29,4 (1–260)
*An. aquasalis*	12	76	44,7	12,8 (1–50)
*A. darlingi*	17	530	18,3	15,9 (1–150)
*An. nuneztovari s.l.*	17	106	24,5	7,3 (1–34)
*An. triannulatus s.l.*	20	260	8,8	3,3 (1–22)
	**IBS-infected mosquitoes**			
*An. albitarsis* s.l.	10	201	40,8	22,5 (1–200)
*A. aquasalis*	9	40	20	5,5 (1–17)
*An. darlingi*	9	217	28,1	11,9 (1–124)
*An. nuneztovari* s.l.	9	43	30,2	11,5 (1–80)
*An. triannulatus s.l.*	10	115	16,5	5,4 (1–29)

Ooc = oocysts, Min = minimal number of oocysts, Max = maximal number of oocysts.

**Figure 2:**
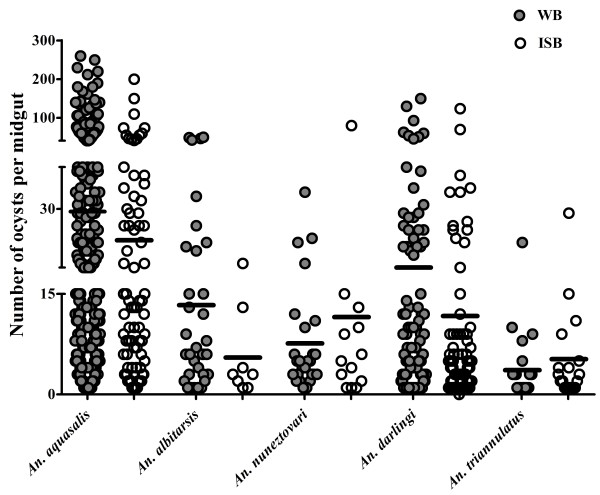
**Comparison of the median numbers of *Plasmodium vivax *** **oocysts of WB-infected and IBS-infected *Anopheles *** **species from the Brazilian Amazon.**

The intensity of infection, measured by the numbers of oocysts per infected mosquitoes, fed on WB and IBS varied significantly among species (K = 48.9, GL = 4, p < 0.001) (Figure 2). The highest number of oocysts was observed in *An. aquasalis* (mean =28.6 ±41.7 S.D, median = 12) and showed statistical difference when compared with all the other species. *Anopheles darlingi* (mean = 15.9 ± 24.3 SD, median = 7) was followed by *An. albitarsis s.l.* (mean = 13.3 ± 14.8 SD, median = 6) and *An. nuneztovari s.l.* (mean = 7.3 ± 8 SD, median = 5). There was no significant difference in the number of oocysts between *An. darlingi*, *An. albitarsis s.l.* and *An. nuneztovari s.l.* However, *An. triannulatus s.l.* had significantly less oocysts than all other studied species (mean = 3.4 ± 4.7 S.D., median = 1).

**Figure 3:**
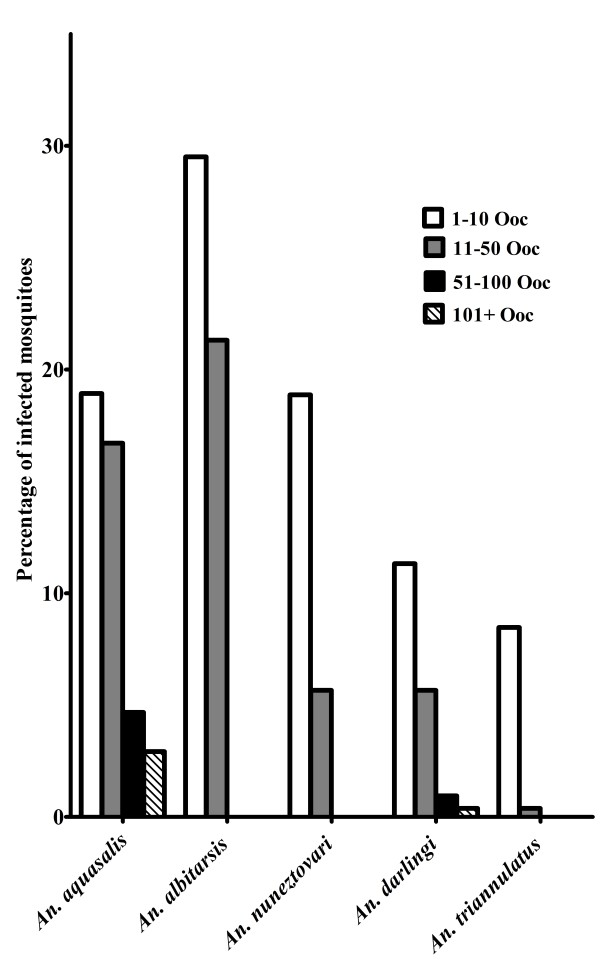
**Percentage of WB-infected *Anopheles *** **mosquitoes from the Brazilian Amazon with different *Plasmodium vivax *** **oocyst densities (Ooc = oocyst numbers).**

To outline the distribution of infection intensities, among WB-infected mosquitoes, we organize the oocyst numbers into four groups, as follows: 1–10, 11–50, 51–100, and more than 100 oocysts. A comparison of the distribution of these groups among individuals within species is illustrated in Figure 3. Sixty-nine per cent of the mosquito specimens did not develop oocysts: the proportion of individuals with zero oocysts was higher in *An. triannulatus* s.l., followed by *An. darlingi*, *An. nuneztovari s.l*., *An. aquasalis,* and *An. albitarsis s.l.* Approximately 16% of individuals from all five species were infected with 1 to 10 oocysts and only 10% were infected with 11 to 50 oocysts, with the lower number of infected individuals observed in *An. triannulatus s.l.* (0.4%) and the higher in *An. albitarsis s.l.* (21.3%). Only *An. darlingi* and *An. aquasalis* had more than 50 oocysts, with 1.3 and 7.6% of the individuals, respectively.

**Figure 4:**
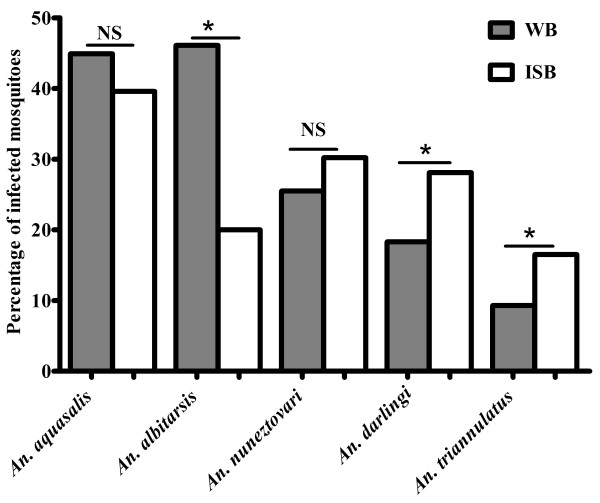
**Comparison of *Plasmodium vivax *** **infection rates of WB-infected and IBS-infected *Anopheles *** species from the Brazilian Amazon (* = Significant, NS = No significant).

Considering all the studied *Anopheles* species as a whole group, the inactivation of factors present in blood serum was not of significant importance to change the infection rate (G = 0.2899, GL = 1, p = 0.585). However, the pattern of the infection rate differed among IBS-infected mosquito species. While the serum inactivation did not cause changes in infection rates on *An. aquasalis* (G = 1.08, GL = 1, p = 0.298) and *An. nuneztovari s.l.* (G = 0.5, GL = 1, p = 0.47), serum inactivation resulted in a 53% increase in infection rates in *An. darlingi* (G = 8.55, GL = 1, p = 0.003), and 87% increase in *An. triannulatus s.l.* (G = 4.4, GL = 1, p = 0.035)*.* Inversely, the infection rate for *An. albitarsis s.l.* decreased by 55% (G = 7.32, GL = 1, p = 0.007) (Figure 4).

In general, the intensity of infection, represented by oocyst numbers, varied among species when inactivated serum was used to infect mosquitoes (IBS-infected mosquitoes). There was no difference in oocyst numbers formed on *An. aquasalis* (U = 1.3, p = 2.44) and *An. nuneztovari s.l.* (Mann–Whitney U = 0.466 p = 0.495). On the other hand, the inactivation slightly increased the oocyst formation on *An. triannulatus* s.l. (U = 7.7, p = 0.006) and decreased in *An. darlingi* (U = 5.01, p = 0.024) and *An. albitarsis s.l.* (U = 8.27; p = 0.004) (Figure 2).

A total of 17 *P. vivax* isolates from malarial patients were used for *An. darlingi* feeding experiments, 13 for *An. albitarsis s.l.,* 17 for *An. nuneztovari s.l*., 20 for *An. triannulatus s.l.* and 29 for *An. aquasalis* (Table 1). Infection rates and range of oocyst numbers per gut varied widely among the different species of WB-infected mosquitoes and IBS-infected mosquitoes. In general, a relationship between the number of gametocytes/500 leukocytes and the infection rate of the mosquitoes (Z = −8.37, p < 0.001) was observed. However, that relationship varied among the species. For *An. darlingi* (Z = −2.9, p < 0.01) and *An. aquasalis* (Z = −4.66, p < 0.001) infection rates increased with an increase in the number of gametocytes. For *An. nuneztovari s.l., An. triannulatus s.l.* and *An. albitarsis s.l.* (Z < 1.02, p > 0.3 in all cases) there was no correlation between those two variables. Also, among infected species, *An. aquasalis* (Spearman rho = 0.255, n = 386, p < 0.01) and *An. darlingi* (rho = 0.518; n = 54, p < 0.01) showed a positive correlation between the number of gametocytes and the number of oocysts formed. The data for all other species did not exhibit a correlation between the quantity of gametocytes and the quantity of oocysts (p > 0.05).

## Discussion

The number of field mosquito specimens available for experimental infection was limited by the seasonality of the species and the malaria transmission peaks in the Amazonas region near to Manaus city. A membrane-feeding assay was used to infect *Anopheles* species. Experimental infection of mosquito vectors can involve direct feeding on the skin of patients or offering blood meal through a membrane-feeding device. Ethical preference tends to lean towards the use of membrane-feeding experiments, in order to minimize the human factor interaction. Most experimental results with Amazon outbred *Anopheles* species have used direct skin feeding on gametocytaemic malarial patients [17,27,64]. These findings are in agreement with other published data where both direct and membrane feeding using blood from *P. vivax*-gametocytaemic patients resulted in *An. darlingi* infection rates between 22 and 23% with a mean oocyst load per infected midgut of 11.5 with a range of 1–175 [65,66]. *Anopheles aquasalis* and *An. albitarsis* s*.l.* had a significantly higher infection rate than *An. darlingi,* considered the main malaria vector in the Brazilian Amazon, and all three species showed high quantity of oocysts, being that the highest one was found in *An. aquasalis*. These results confirm that the membrane-feeding assay is as efficient as direct feeding on human skin when it comes to the study of *Plasmodium* infection on mosquito vectors.

*Anopheles darlingi* in Amazon area is more abundant during the late wet season and early dry season, while the other species are more abundant during the early dry season [44,67]. Similar situation occurs in Manaus region (personal observation). During the time period when *An. darlingi* was in high abundance, *An. albitarsis s.l.* and *An. nuneztovari s.l.* were in low abundance. When the abundance of *An. darlingi* was low, *An. albitarsis s.l.* and *An. nuneztovari s.l.* abundance increased. This dynamics in species succession is a very important factor for maintenance of malaria transmission, and can present difficulties for malaria control in this region due to susceptibility of all studied species to *P. vivax* infection [68]*.*

Here, it was demonstrated that *An. darlingi, An. albitarsis s.l., An. nuneztovari s.l.,* and *An. triannulatus s.l.* field populations, and the laboratory-colonized *An. aquasalis* are susceptible to *P. vivax* under laboratory conditions. All of the studied species might be sporadic competent vectors in nature, although there was a generalized high proportion of uninfected mosquitoes. However, infection rates were much higher than those reported in nature for the five species examined. As determined by the ELISA technique, based on the use of species-specific anti-sporozoite monoclonal antibodies, Amazonian mosquito populations had different *P. vivax* infection rates: *An. darlingi* ranged from 0.3 to 9.3%; *An. albitarsis s.l.* from 0.4 to 5.2%; *An. nuneztovari s.l.* from 0.3 to 1.1%; *An. triannulatus* s*.l.* 0.2% and *An. aquasalis* from 0.3 to 1.3% [15,18,20-25,27,69-71]. Since this study used blood infected with gametocytes that was offered to mosquitoes, higher infection rates were expected, but variable infectivity in the same setting of gametocytaemia was observed. Differences in infectivity of the different blood samples could be due to a combination of variables, such as, gametocyte maturity, gametocyte gender ratio, different *P. vivax* genotypes, immune factors in patient sera, and host response mechanisms, all of which could alter gametocyte infectivity [27,48,72-74].

The number of oocysts has little importance in malaria epidemiology since most infected mosquitoes found in nature only possesses a few oocysts [75]. The degree of anthropophily in nature is probably the most important factor to determine vector capacity [76]. In sites around Manaus, Brazil, *An. darlingi* is known to be strongly anthropophilic and endophilic, and population of this species occurs through the year. *Anopheles albitarsis s.l.* was shown to be a very susceptible species to *P. vivax*, and these results agree with results obtained in other Brazilian Amazonian states of Roraima, Pará, Amapá and Rondônia [15,16,19,70]. However, Klein and collaborators considered *An. albitarsis s.l.* a dubious malaria vector because of the low number of oocysts, zoophilic behavior and seasonal distribution [44]. Like *An. darlingi* and *An. albitarsis s.l.*, *An. aquasalis* showed a high susceptibility to *P. vivax*. The high level of susceptibility of *An. aquasalis* to *P. vivax* indicates the value of using this species in studying New World malarial parasite vector-interaction. Unlike *An. darlingi*, *An. aquasalis* is well adapted to colonization in the laboratory.

*Anopheles nuneztovari s.l*. also showed a high infection rate, although not statistically different to *An. darlingi,* it was significantly different to *An. albitarsis s.l..* Oocyst numbers in *An. nuneztovari s.l.* were lower than in *An. darlingi* and *An. albitarsis s.l.*, although the mean number of oocysts was not significantly different between these species. *Anopheles nuneztovari s.l.* is considered an important malaria vector in some South American countries [30,31]; however, for others, this species is not considered a malaria vector because natural infections are rarely observed and when they are, infection rates are very low [18,23,24]. The results presented here clearly suggest the high potential of *An. nuneztovari s.l.* as a vector of malaria, which could be considered a risk depending on its density in a given area. In the Manaus area for example, *An. nuneztovari s.l.* is probably an important species for malaria transmission, because of its high feeding and infection rates observed in laboratory. The population of this species was found in high densities in localities around Manaus where *An. darlingi* is also abundant [18]. On the other hand, although *An. triannulatus s.l.* became infected with *P. vivax* in the experiments described here, this species had significantly the lowest infection rates and mean number of oocysts compared with the other species studied. These observations reinforce the conclusion that *An. triannulatus s.l.* is not an important malaria vector in the Amazon region [15,16,44,68].

Except for *An. albitarsis s.l.* and *An. aquasalis,* mosquito infection rates were increased after blood serum inactivation. Blood serum factors have been shown to influence the ability of *P. vivax* gametocytes to infect mosquitoes in experiments in which patient plasma was replaced with *P. vivax*-naïve sera or plasma [46,66]. Infection in these two mosquito species does not appear to be strongly related to host immune factors. Resistance to *Plasmodium* infection in *An. albitarsis s.l*. and *An. aquasalis* may be more strongly associated with intrinsic factors related to the mosquito’s own immune system, which could respond more effectively to destroy *Plasmodium* infections. Future studies should include evaluation of *P. vivax* strain variability on mosquito susceptibility and both intra- and interspecific variation in mosquito immune responses to *Plasmodium* infection.

The results also indicate that the quantity of gametocytes are positively correlated with the infection rate and the number of oocysts formed, but when results were analyzed by individual species only *An. darlingi* and *An. aquasalis* had a positive correlation between the quantity of gametocytes and the other two variables. However, according to Klein and collaborators there was no correlation between the number of *P. falciparum* gametocytes and the mean number of oocysts formed in *An. darlingi*, although, in general, low numbers of circulating gametocytes resulted in few infected mosquitoes [17]. Distinct results could be related to observational methods for verifying the quantity of gametocytes, as determined by light microscopy, which does not predict *P. vivax* transmission to mosquitoes [77].

## Conclusion

Development of novel malaria control strategies includes methods aimed at disrupting parasite development in the mosquito vector. Studying natural vector-parasite interactions, as opposed to model systems, is critical to the development of strategies that can ultimately be used in the field. These studies are made difficult in cases where mosquito vectors cannot be colonized in the laboratory. Such is the case with many of the vectors in South America, including the main vector *An. darlingi*, which has not been colonized after several efforts by distinct research groups (personal observation). Only *An. aquasalis*, the main vector in coastal Brazil, has been established and maintained in the laboratory. This study established baseline data for key transmission parameters showing that in laboratory, *P. vivax* infection of colonized *An. aquasalis* had an infection rate of 44.8% with a mean oocyst count of 29 per infected individual. It is possible now to begin using this system to explore mosquito immune response to *P. vivax* infection [57-59].

## Competing interests

The authors declare that they have no competing interests.

## Authors’ contributions

CMRV, FACP, RS, PFPP, NFCS, WPT, and MVGL conceived and designed the experiments; CMRV, KMC, RS, FACP, EVS, and JBPL: field collection and laboratory procedures; CMRV and TJI: data analysis; CMRV, PFPP, TJI, WMM, FACP, WPT, MVGL, and NFCS: data interpretation and manuscript preparation. This manuscript is a part of the PhD thesis developed by CMRV and supervised by PFPP. PFPP is a senior visiting researcher at the Foundation of the Tropical Medicine Dr. Heitor Vieira Dourado in Manaus (FMT-HVD). PFPP, NFCS and MVGL are productivity fellows of the Brazilian Council for Scientific and Technological Development (CNPq). All authors read and approved the final version of this article.
